# Knowledge, attitudes, and practices of parents regarding childhood weight management and associated factors in Southwest China: a cross-sectional study

**DOI:** 10.3389/fpubh.2026.1907801

**Published:** 2026-09-24

**Authors:** Liangjia Wei, Rufei Wei, Wenjing Huang, Jiahao Lian, Yang Li, Min Zhou, Jianying Liao, Rui Guo, Shengkui Tan

**Affiliations:** Guangxi Zhuang Autonomous Region Gastrointestinal Microecology Intelligent Biomedical Engineering Research Center,Youjiang Medical University for Nationalities, Baise, Guangxi, China

**Keywords:** attitudes, parents, practices, school-age children, weight management

## Abstract

**Objectives:**

Following the implementation of the Weight Management Year initiative in China, parents’ knowledge, attitudes, and practices (KAP) regarding childhood weight management remain unclear. This study aimed to assess weight management-related KAP and the factors associated with weight management practices among parents of school-age children in Southwest China.

**Methods:**

A cross-sectional survey was conducted from November to December 2025 among parents of school-age children in Southwest China. Parents completed a structured online questionnaire assessing sociodemographic characteristics and KAP related to childhood weight management. Factors associated with poor weight management practices were identified using multivariable logistic regression. Mediation analysis was employed to examine the potential mediating role of attitudes in the pathway from weight management policy, and knowledge to practices.

**Results:**

Among the 572 participants, 76.2% were female and over half (51.9%) reported awareness of China’s weight management policy. The mean scores for knowledge, attitudes, and practices were 7.83 ± 3.03 (range: 0–11), 34.81 ± 5.46 (range: 13–45), and 43.14 ± 6.38 (range: 21–55), respectively. Rural residence (aOR = 1.69, 95% CI: 1.09–2.66), uncontrolled eating (aOR = 1.70, 95% CI: 1.13–2.57), lack of awareness of China’s 2024 weight management policy (aOR = 1.98, 95% CI: 1.31–3.00), and negative attitudes (aOR = 5.32, 95% CI: 3.45–8.28) were identified as significant correlates of poor weight management practices. Weight management attitudes mediated the effects of policy awareness (mediating proportion: 27.7%, 95% CI: 10.8–57.4) and knowledge (mediating proportion: 69.2, 95% CI: 36.4–189.5) on practices.

**Conclusion:**

These findings highlight the urgent need for targeted public health interventions that go beyond merely disseminating weight management knowledge, and actively address and reshape parental attitudes—particularly among rural residents, fathers, and children with overweight or obesity—thus effectively translating national guidelines into improved weight management practices at the household level.

## Introduction

Childhood obesity is a pressing global public health challenge. A report released by the United Nations Children’s Fund in 2025 revealed that 188 million children and adolescents aged 5–19 were living with obesity, representing a global obesity rate of 9.4% (1). According to the World Obesity Atlas 2024 published by the World Obesity Federation, 37% of the population aged 5–19 in China were overweight or obese in 2020, representing over 97 million individuals. This proportion is projected to rise to 72% by 2035, with the total number reaching 130 million (2). The prevalence of overweight and obesity has been rising for decades, with the increase being most pronounced among primary school children (3). Obesity was strongly associated with childhood health issues, such as non-alcoholic fatty liver disease, metabolic syndrome, and precocious puberty (4–6). Given that children with obesity were more likely to maintain this phenotype into later life (7), which is linked to an increased risk of chronic diseases in adulthood. Therefore, effective weight management during childhood is of critical importance.

Childhood obesity was associated with multiple factors, including biological (e.g., prenatal exposure, epigenetic mechanisms), behavioral (e.g., poor dietary habits, inadequate physical activity, excessive screen time), and family and social determinants (e.g., early feeding practices, household food environments) (8–11). Among these, family factors—particularly those involving parents—play a direct and modifiable role in shaping children’s weight status. In efforts to prevent and control childhood obesity, parents therefore serve as the primary architects of their children’s weight-related health. For example, a study found that parents’ socioeconomic status was associated with obesity in school-aged children, primarily through the mediating role of their weight-related knowledge, attitudes, and practices (12). A meta-analysis by Robbins et al. demonstrated that parental-involved physical activity and nutrition interventions significantly improved weight-related outcomes, including BMI, body fat percentage, and waist circumference, in young adolescents (13). These findings underscore the crucial role of parental involvement in addressing childhood obesity.

Despite various weight management interventions, long-term adherence remains limited, often due to insufficient parental engagement, highlighting the need to identify the factors that shape parents’ involvement in childhood weight management. The Knowledge, Attitude, and Practice (KAP) framework offers a valuable analytical lens for this purpose (14). A cross-sectional study from Iran, using multinomial logistic regression analysis, showed that mothers’ insufficient knowledge and negative attitudes toward food security were associated with poorer child anthropometric indicators, consequently increasing the risk of childhood obesity (15). Similarly, another cross-sectional descriptive study, using inferential statistical analyses, found that mothers’ attitudes toward their child’s weight and diet were significantly related to this risk (16). Fathers also play a role in childhood obesity prevention. For instance, a cross-sectional study in Saudi Arabia, using Spearman’s correlations and descriptive statistics, found that higher paternal self-efficacy was associated with less controlling feeding practices and greater perception of child overweight (17). While these studies collectively highlight the importance of parental cognitive and behavioral factors in shaping childhood obesity risk, most have relied on cross-sectional designs with descriptive or regression-based analyses, which primarily establish associations rather than quantify the underlying mediating mechanisms. This gap is significant, particularly from a behavioral intervention perspective, as understanding whether attitudes mediate the link between knowledge and practice is essential for designing effective, targeted strategies. In the absence of quantitative mediation evidence, interventions risk overemphasizing knowledge dissemination while overlooking the attitudinal internalization that underpins sustainable behavioral change. Consequently, the extent to which parental knowledge and attitudes translate into practices—and subsequently influence child weight outcomes—remains insufficiently understood from a quantitative mediation perspective.

In response to the growing obesity challenge, China has prioritized obesity prevention at the national level. The Chinese government launched the “Weight Management Year” campaign to promote healthy lifestyles, enhance weight management skills, and advance chronic disease prevention (18). However, in this context, little is known about Chinese parents’ perceptions and engagement in childhood weight control, despite their central role as primary caregivers. Notably, the mediating pathways through which parental knowledge and policy awareness translate into weight management practices have not been quantitatively examined in the Chinese context. Therefore, guided by the KAP framework, this study aimed to assess parental weight management-related knowledge, attitudes, and practices and to identify associated factors among parents of school-age children in Southwest China. Specifically, we sought to (1) describe parental KAP levels, (2) examine associated factors, and (3) quantitatively estimate the mediating effects of parental attitudes on the relationship between knowledge or policy awareness and practices. By providing quantitative estimates of mediation effects, this study offers a new, empirically grounded perspective that may inform the design of future family-centered weight management interventions targeting school-age children in China.

## Methods

### Study design, and participants

A cross-sectional survey was conducted from November to December 2025 at two public primary schools in Hechi City, Guangxi Zhuang Autonomous Region, Southwest China. Prior to participant recruitment, the research team conducted standardized training sessions for all homeroom teachers of grades 1–6 at the participating schools. The training covered the study’s objectives, the survey content, and detailed instructions on how to guide parents to complete the online questionnaire accurately. Participants were then recruited by a teacher-facilitated, convenience sampling approach: teachers distributed the survey link to parents via established class communication channels (e.g., WeChat groups), and the questionnaire was completed online by either the mother or the father of each child. Written informed consent was obtained from all participants prior to data collection. All personal information was kept confidential throughout the research process. This study has been reviewed and approved by the Ethics Committee of Youjiang Medical University for Nationalities (No. 2025091501). All participants were aware of the content and purpose of this study and agreed to provide written informed consent before this survey.

The inclusion criteria were: (1)Biological or adoptive parents of a child enrolled in grades 1–6 at the participating schools (if multiple children were eligible, one was randomly designated as the index child based on the closest birthday to the survey date); (2) Acts as the child’s primary caregiver and a key decision-maker in their daily care; (3) age ≥ 18 years; (4) Able to read and comprehend the survey language (Chinese); (5) providing written informed consent. The exclusion criteria were:(1) declining to participate; (2) Inability to comprehend the questionnaire content or incomplete questionnaire responses.

### Sample size

The sample size was calculated using the formula for estimating a proportion in cross-sectional studies. With a 95% confidence level (*Z* = 1.96), an expected proportion of 0.5, and a margin of error of 5% (*δ* = 0.05), the minimum sample size was 385. Accounting for a 15% non-response rate, the adjusted target sample size was 453.

### Questionnaire

The questionnaire was designed based on expert discussions and a comprehensive review of relevant literature (19–21)^.^ In a pilot test, 150 valid responses were collected. Reliability analysis demonstrated a Cronbach’s alpha coefficient of 0.851, indicating good internal consistency of the instrument.

In Section I, detailed sociodemographic and health-related information was collected from the participating parents. All child-related data, including age, sex, current body weight status (categorized as underweight, normal weight, or overweight/obese), and history of chronic diseases, were provided by parental report. For the parent, data included: age, sex, ethnicity, residence, education, occupation, family monthly income, personal history of chronic diseases, eating behaviors (cognitive restraint, emotional eating, and uncontrolled eating), and awareness of China’s 2024 national weight management policy. To assess eating behaviors, we utilized the validated Polish version of the Three-Factor Eating Questionnaire (TFEQ-13), which comprises three subscales: cognitive restraint, emotional eating, and uncontrolled eating. Responses were recorded on a 4-point Likert scale, ranging from strongly disagree to strongly agree. The Emotional Eating subscale consists of 4 items, which measure the tendency to overeat in response to negative emotions or stress (score range: 4–16). The controlled eating subscale, also with 4 items, assesses an individual’s ability to effectively regulate hunger and manage overeating through self-control(range: 4–16). The Cognitive Restraint subscale is composed of 5 items that measure deliberate efforts to restrict food consumption for weight and body image control (range: 5–20).

Knowledge, attitudes, and practices were assessed in Sections II to IV, respectively. Detailed item descriptions are provided in Supplementary Tables S11–S13. The knowledge dimension comprised 11 items with “true,” “false,” or “unsure” response options. For the items, a correct “true” response was scored 1 point, while “false” or “unsure” responses were scored 0. For analysis, we combined ‘true’ and ‘false’ into one category. However, four items were reverse-scored, meaning a correct “no” response earned 1 point, and “yes” or “unsure” responses earned 0. These items pertained to the following statements: (a) during medical nutrition therapy for weight loss, there is no need to supplement micronutrients. (b) Maintaining regular sleep patterns is not a component of behavioral intervention for weight management. (c) Overweight/obesity can be cured by surgery alone. (d) Preventing obesity-related diseases is not a clinical goal of weight management for overweight/obese patients. Total scores for the knowledge dimension ranged from 0 to 11. The attitude dimension comprised 9 items measured on a 5-point Likert scale, ranging from 1 (“Strongly Disagree”) to 5 (“Strongly Agree”). For most items, higher scores indicated more positive attitudes; however, three items were reverse-scored (e.g., “Perceiving a child’s abnormal weight as a normal developmental phase,” “Believing that eating less alone ensures weight loss,” and “Believing that taking weight-loss pills makes it unnecessary to modify diet, physical activity, or behavioral routines.”) were reverse-scored. Total attitude scores ranged from 13 to 45. The practice dimension contained 11 items scored on a 5-point frequency scale from 1 (never) to 5 (always), One item (“using food as a reward for your child”) was reverse-scored. Total practice scores ranged from 21 to 55. The total score for each dimension was calculated by summing the corresponding item scores. Participants who scored ≥70% of the maximum possible score in a dimension were categorized as having “adequate knowledge,” “positive attitudes,” or “positive practice,” respectively (22).

### Statistical analysis

All data were double-entered into EpiData version 3.1 software for Windows (Odense, Denmark) and R version 4.3.1 for statistical analysis. Figures were processed and edited using Adobe Photoshop 2020. Categorical variables were summarized as frequencies and percentages. Differences in these variables across practice groups were assessed using Pearson’s chi-square test. Multivariate logistic regression was used to identify the correlates of weight management practices. To address the potential selection bias inherent in the convenience sampling design—particularly regarding differences in characteristics between the positive- and negative-practice groups—we adopted a two-step analytical approach. First, we quantified the comparability of the two groups using absolute standardized mean differences (SMDs) across all sociodemographic variables, with |SMD| < 0.1 indicating negligible imbalance and values between 0.1 and 0.2 suggesting mild imbalance. The largest absolute SMD between groups was 0.199 (family income ≥5,000 CNY/month), suggesting that the two groups were reasonably comparable. Second, to further reduce potential selection bias and improve the representativeness of the analytic sample for the target population, we re-estimated the associations between each of the three key exposures (policy awareness, uncontrolled eating, and attitudes) and weight management practices using propensity-score overlap weights (PSOW). PSOW targets the average treatment effect in the overlap population, approximately balances all covariate means under a logistic propensity model, and avoids the extreme weights that can arise with inverse-probability weighting. Propensity scores were modeled based on all child and parent sociodemographic characteristics. Then, we compared the unweighted and PSOW-weighted logistic regression estimates to assess the robustness of the observed associations. Moreover, stratified analyses were also conducted by parental age, gender, residence, and school-aged children’s BMI.

To further examine potential mediating pathways, a mediation analysis was performed using the mediation package in R. First, a logistic regression model was fitted to assess the effect of knowledge (or weight management policy) on the binary mediator, weight-management attitudes, while adjusting for covariates. Second, another logistic regression model was specified to assess the effects of both knowledge (or weight management policy) and weight management attitudes on the weight management practice, with the same covariates. A bootstrap-based mediation analysis (5,000 simulations) was then conducted to estimate the indirect (mediated) and direct effects, along with their 95% confidence intervals. The proportion mediated was calculated as the ratio of the indirect effect to the total effect. The significance of the mediation effect was evaluated using the bootstrap confidence interval approach. To assess the robustness of the mediation findings to potential selection bias, we repeated the mediation analysis using PSOW-weighted samples. A two-sided *p*-value <0.05 was considered statistically significant.

## Results

### Characteristics of participants

A total of 572 participants were included in this study. The majority of parents were female (76.2%) and aged ≥35 years (68.2%). Most families resided in rural areas (65.9%), and slightly over half of them (51.9%) reported being aware of China’s 2024 national “Weight Management Year” policy. The mean scores for parental knowledge, attitudes, and practices regarding childhood weight management were 7.83 ± 3.03 (range: 0–11), 34.81 ± 5.46 (range: 13–45), and 43.14 ± 6.38 (range: 21–55), respectively. In terms of child characteristics, nearly half (47.2%) were aged 8–9 years, and 27.8% were classified as overweight or obese (Table 1).

**Table 1:** Characteristics of participants.

Variables	Total (*n* = 572)	Percentage (95% CI)
Child characteristics		
Age (years)		
6–7	83	14.5 (11.8, 17.7)
8–9	270	47.2 (43.1, 51.4)
10–12	219	38.3 (34.3, 42.4)
Sex		
Male	311	54.4 (50.2, 58.5)
Female	261	45.6 (41.5, 49.8)
BMI status		
Underweight	144	25.2 (21.7, 29.0)
Normal weight	269	47.0 (42.9, 51.2)
Overweight or obese	159	27.8 (24.2, 31.7)
Chronic disease		
No	525	91.8 (89.1, 93.8)
Yes	47	8.2 (6.2, 10.9)
Parent characteristics		
Age (years)		
18–34	182	31.8 (28.0, 35.8)
≥35	390	68.2 (64.2, 72.0)
Sex		
Male	136	23.8 (20.4, 27.5)
Female	436	76.2 (72.5, 79.6)
Ethnicity		
Han	71	12.4 (9.9, 15.5)
Minority	390	68.2 (64.2, 72.0)
Other	111	19.4 (16.3, 22.9)
Residence		
Urban	195	34.1 (30.2, 38.2)
Rural	377	65.9 (61.8, 69.8)
Education		
Primary school and below	33	5.8 (4.1, 8.1)
Junior school	175	30.6 (26.9, 34.6)
High school	127	22.2 (18.9, 25.9)
College and above	237	41.4 (37.4, 45.6)
Occupation		
Enterprise worker	92	16.1 (13.2, 19.4)
Government employee	98	17.1 (14.2, 20.5)
Self-employed	193	33.7 (29.9, 37.8)
Unemployed	35	6.1 (4.4, 8.5)
Farmer	98	17.1 (14.2, 20.5)
Other	56	9.8 (7.5, 12.6)
Family income (CNY/month)		
<2,000	107	18.7 (15.6, 22.2)
2,001 ~ 3,000	168	29.4 (25.7, 33.3)
3,001 ~ 4,999	167	29.2 (25.5, 33.1)
≥5,000	130	22.7 (19.4, 26.4)
Chronic disease		
No	541	94.6 (92.3, 96.2)
Yes	31	5.4 (3.8, 7.7)
Cognitive restraint eating		
No	335	58.6 (54.4, 62.6)
Yes	237	41.4 (37.4, 45.6)
Emotional eating		
No	250	43.7 (39.6, 47.9)
Yes	322	56.3 (52.1, 60.4)
Uncontrolled eating		
No	328	57.3% (95% CI: 53.2–61.4)
Yes	244	42.7% (95% CI: 38.6–46.8)
Awareness of China’s 2024 weight management policy		
No	275	48.1 (43.9, 52.3)
Yes	297	51.9 (47.7, 56.1)
Knowledge score (Mean ± SD)	7.83	7.83 ± 3.03
Attitudes score (Mean ± SD)	34.81	34.81 ± 5.46
Practice score (Mean ± SD)	43.14	43.14 ± 6.38

### Distribution of responses in knowledge, attitudes, and practices

Regarding knowledge items, two items had notably high rates of false responses. For the item “The causes of overweight/obesity includes genetic and environmental factors,” 40.6% of participants responded false. For the reverse-scored item “There is no need to supplement with micronutrients during medical nutritional weight loss,” 53.8% responded false (indicating incorrect knowledge). Regarding the attitude items, only 15.0% of parents strongly disagreed with the statement that “Abnormal weight in children at the current stage (such as underweight, overweight, or obesity) is just a normal part of growing up.” For the belief that “Children can lose weight simply by eating less,” 18.4% of parents strongly disagreed. Furthermore, only 21.0% of parents strongly agreed that they were confident in managing their child’s weight. Only 6.5% of parents strongly agreed that implementing scientific weight management practices—such as dietary modification and physical activity—was easy to incorporate into their daily lives. Regarding the practice dimension, among all items, only “Encourages the child to go to bed early every day” was reported by a majority of parents (53.8%) (Supplementary Tables S11–S13).

### Characteristic differences by parental weight management practice status

As shown in Table 2, parents with poor weight management practices differed significantly from those with positive practices across several key characteristics. They had a higher proportion reporting uncontrolled eating (51.0% vs. 39.7%, *p* = 0.021), lower awareness of the national “Weight Management Year” policy (62.4% vs. 43.0%, *p* < 0.001), a greater proportion with inadequate knowledge (48.3% vs. 31.7%, *p* < 0.001), and a markedly higher frequency of negative attitudes (47.0% vs. 14.2%, *p* < 0.001). No other characteristics showed statistically significant differences between the two groups (*p* > 0.05).

**Table 2:** Comparison of characteristics by parental weight management practice level.

Variables	Positive practice	Negative practice	*p* value
	*n* = 423	*n* = 149	
Child characteristics			
Age (years)			0.813
6–7	59 (13.9)	24 (16.1)	
8–9	201 (47.5)	69 (46.3)	
10–12	163 (38.5)	56 (37.6)	
Sex			0.776
Male	228 (53.9)	83 (55.7)	
Female	195 (46.1)	66 (44.3)	
BMI status			0.508
Underweight	104 (24.6)	40 (26.8)	
Normal weight	205 (48.5)	64 (43.0)	
Overweight or obese	114 (27.0)	45 (30.2)	
Chronic disease			0.434
No	391 (92.4)	134 (89.9)	
Yes	32 (7.6)	15 (10.1)	
Parent characteristics			
Age (years)			1.0
18–34	135 (31.9)	47 (31.5)	
≥35	288 (68.1)	102 (68.5)	
Sex			0.174
Male	94 (22.2)	42 (28.2)	
Female	329 (77.8)	107 (71.8)	
Ethnic**ity**			0.333
Han	53 (12.5)	18 (12.1)	
Minority	282 (66.7)	108 (72.5)	
Other	88 (20.8)	23 (15.4)	
Residence			0.287
Urban	150 (35.5)	45 (30.2)	
Rural	273 (64.5)	104 (69.8)	
Education			0.696
Primary school and below	23 (5.4)	10 (6.7)	
Junior school	125 (29.6)	50 (33.6)	
High school	97 (22.9)	30 (20.1)	
College and above	178 (42.1)	59 (39.6)	
Occupation			0.356
Enterprise worker	63 (14.9)	29 (19.5)	
Government employee	76 (18.0)	22 (14.8)	
Self-employed	137 (32.4)	56 (37.6)	
Unemployed	29 (6.9)	6 (4.0)	
Farmer	77 (18.2)	21 (14.1)	
Other	41 (9.7)	15 (10.1)	
Family income (CNY/month)			0.225
<2,000	79 (18.7)	28 (18.8)	
2,001 ~ 3,000	119 (28.1)	49 (32.9)	
3,001 ~ 4,999	120 (28.4)	47 (31.5)	
≥5,000	105 (24.8)	25 (16.8)	
Chronic disease			0.858
No	401 (94.8)	140 (94.0)	
Yes	22 (5.2)	9 (6.0)	
Cognitive restraint eating			0.311
No	242 (57.2)	93 (62.4)	
Yes	181 (42.8)	56 (37.6)	
Emotional eating			0.302
No	179 (42.3)	71 (47.7)	
Yes	244 (57.7)	78 (52.3)	
Uncontrolled eating			0.021
No	255 (60.3)	73 (49.0)	
Yes	168 (39.7)	76 (51.0)	
Awareness of China’s 2024 weight management policy			<0.001
No	182 (43.0)	93 (62.4)	
Yes	241 (57.0)	56 (37.6)	
Knowledge			<0.001
Inadequate	134 (31.7)	72 (48.3)	
Adequate	289 (68.3)	77 (51.7)	
Attitude			<0.001
Negative	60 (14.2)	70 (47.0)	
Positive	363 (85.8)	79 (53.0)	

### Factors associated with poor weight management practices

Multivariable logistic regression analysis identified several independent correlates of poor weight management practices. Residing in rural areas was associated with significantly higher odds compared to urban residence (aOR = 1.69, 95% CI: 1.09–2.66). Parents exhibiting uncontrolled eating behaviors also had increased odds (aOR = 1.70, 95% CI: 1.13–2.57). Lack of awareness of China’s 2024 weight management policy was another significant correlate (aOR = 1.98, 95% CI: 1.31–3.0). Most prominently, holding a negative attitude toward weight management was the correlate, with odds approximately 5.32 times higher than those with a positive attitude (aOR = 5.32, 95% CI: 3.45–8.28) (Table 3).

**Table 3:** Factors associated with poor weight management practices among parents.

Variables	Univariable	Multivariable
	aOR (95% CI) *p*	aOR (95% CI) *p*
Child characteristics		
Age (years)		
6–7	ref	
8–9	0.84 (0.49–1.48) 0.544	
10–12	0.84 (0.48–1.5) 0.557	
Sex		
Male	ref	
Female	0.93 (0.64–1.35) 0.704	
BMI status (parent-reported)		
Underweight	ref	
Normal weight	0.81 (0.51–1.29) 0.374	
Overweight or obese	1.03 (0.62–1.7) 0.919	
Chronic disease		
No	ref	
Yes	1.37 (0.7–2.56) 0.34	
Parent characteristics		
Age (years)		
18–34	ref	
≥35	1.02 (0.68–1.52) 0.933	
Sex		
Male	ref	
Female	0.73 (0.48–1.12) 0.142	
Ethnicity		
Han	ref	
Minority	1.13 (0.64–2.06) 0.684	
Other	0.77 (0.38–1.57) 0.466	
Residence		
Urban	ref	ref
Rural	1.27 (0.85–1.91) 0.245	1.69 (1.09–2.66) 0.022
Education		
Primary school and below	ref	
Junior school	0.92 (0.42–2.15) 0.84	
High school	0.71 (0.31–1.72) 0.431	
College and above	0.76 (0.35–1.76) 0.505	
Occupation		
Enterprise worker	ref	
Government employee	0.63 (0.33–1.2) 0.16	
Self-employed	0.89 (0.52–1.53) 0.666	
Unemployed	0.45 (0.15–1.14) 0.111	
Farmer	0.59 (0.31–1.13) 0.116	
Other	0.79 (0.37–1.64) 0.541	
Family income (CNY/month)		
<2,000	ref	
2,001 ~ 3,000	1.16 (0.68–2.02) 0.589	
3,001 ~ 4,999	1.11 (0.64–1.92) 0.721	
≥5,000	0.67 (0.36–1.24) 0.204	
Chronic disease		
No	ref	
Yes	1.17 (0.5–2.53) 0.697	
Cognitive restraint eating		
No	ref	
Yes	0.81 (0.55–1.18) 0.268	
Emotional eating		
No	ref	
Yes	0.81 (0.55–1.17) 0.259	
Uncontrolled eating		
No	ref	ref
Yes	1.58 (1.09–2.3) 0.017	1.7 (1.13–2.57) 0.011
Awareness of China’s 2024 weight management policy		
Yes	ref	ref
No	2.2 (1.5–3.24) < 0.001	1.98 (1.31–3.0) 0.001
Knowledge		
Adequate	ref	
Inadequate	2.02 (1.38–2.95) < 0.001	
Attitude		
Positive	ref	ref
Negative	5.36 (3.52–8.21) < 0.001	5.32 (3.45–8.28) < 0.001

To assess the robustness of the associations between the three key exposures (policy awareness, uncontrolled eating, and attitudes) and poor weight management practices, we compared the unweighted and PSOW-weighted logistic regression estimates. The direction, magnitude, and statistical significance of the associations remained consistent after weighting (Supplementary Table S1).

### Mediation analysis

The mediation analysis revealed that unawareness of the weight management policy exerted a significant total effect on poor weight management practices (*β* = 0.15, 95% CI: 0.07–0.22). This effect was decomposed into a significant direct path (*β* = 0.11, 95% CI: 0.03–0.18) and a significant indirect effect mediated by weight management attitudes (*β* = 0.04, 95% CI: 0.01–0.07), with attitudes accounting for 27.7% (95% CI: 10.8–57.4) of the total effect (Table 4).

**Table 4:** Direct and indirect associations of weight management policy with weight management practices mediated by weight management attitude, before and after PSOW weighting.

Variables	Unweighted β (95% CI)	PSOW-weighted β (95% CI)
Total association	0.15 (0.07, 0.22)	0.14 (0.07, 0.22)
Direct association	0.11 (0.03, 0.18)	0.10 (0.03, 0.18)
Indirect association (via attitude)	0.04 (0.01, 0.07)	0.04 (0.01, 0.07)
Proportion mediated, %	27.7 (10.8, 57.4)	28.2 (9.8, 59.0)

Adjusted for child characteristics (age, sex, current BMI status, chronic disease) and parent characteristics (age, sex, ethnicity, residence, education, occupation, family monthly income, chronic disease).

Additionally, a significant mediating effect of weight management knowledge on practices was observed. This effect was primarily explained by a significant indirect pathway via weight management attitudes (*β* = 0.08, 95% CI: 0.05–0.12), whereas the direct effect was not significant (*β* = 0.04, 95% CI: −0.04–0.12). The proportion of the total effect mediated via attitudes was 69.2% (95% CI: 36.4–189.5) (Table 5).

**Table 5:** Direct and indirect associations of weight knowledge with weight management practices mediated by weight management beliefs, before and after PSOW weighting.

Variables	Unweighted β (95% CI)	PSOW-weighted β (95% CI)
Total association	0.12 (0.04, 0.20)	0.11 (0.03, 0.20)
Direct association	0.04 (−0.04, 0.12)	0.03 (−0.05, 0.11)
Indirect association (via attitude)	0.08 (0.05, 0.12)	0.08 (0.05, 0.12)
Proportion mediated, %	69.2 (36.4, 189.5)	70.6 (35.7, 227.3)

Adjusted for child characteristics (age, sex, current BMI status, chronic disease) and parent characteristics (age, sex, ethnicity, residence, education, occupation, family monthly income, chronic disease, weight management policy).

Additionally, the results remained consistent after PSOW-weighting, which further supports the robustness of the mediating role of parental attitudes in translating knowledge and policy awareness into weight management practices (Tables 4, 5).

### Stratified analyses

Stratified analyses revealed that a negative attitude was a robust correlate across all subgroups, its association was substantially stronger among fathers (aOR = 11.31) and children with overweight or obesity (aOR = 13.11). Similarly, Lack of awareness of China’s 2024 weight management policy remained a significant risk factor in most subgroups, with notably elevated odds among urban-dwelling parents (aOR = 3.58) and children with overweight or obesity (aOR = 3.59). The association between uncontrolled eating behaviors and poor practices was not uniformly significant across subgroups. It was most pronounced among fathers (aOR = 4.12) (Supplementary Tables S2–S10).

### Parents’ preferred information channels for childhood weight management

The survey revealed a clear hierarchy in parents’ preferred channels for obtaining childhood weight management information. School-based lectures emerged as the predominant choice, selected by 76.7% (*n* = 439) of respondents. This was followed by health education provided by authoritative institutions (49.3%, *n* = 282), professional doctors (41.4%, *n* = 237), social media (38.6%, *n* = 221), books or magazines (38.6%, *n* = 221), and peer communication (conversations with other parents) (30.9%, *n* = 177). Other information channels were reported by 5.1% (*n* = 29) of respondents (Supplementary Figure S1).

## Discussion

In the context of China’s “Weight Management Year” policy, this study conducted a cross-sectional survey to examine KAP and its associated factors among parents of school-aged children in southwestern China. We found that parental attitude showed the strongest association with child weight management practices, and that these practices were also associated with rural residence, uncontrolled eating behaviors, and lack of awareness of the weight management policy. Further analysis revealed that attitude mediated the effects of knowledge and policy awareness on practices. These findings help identify key intervention leverage points among vulnerable subpopulations and inform targeted weight management strategies for school-aged children.

Specifically, compared with parents holding positive attitudes, those with negative attitudes were more likely to engage in poor practices, exhibiting a substantially larger effect size than other significant determinants. Stratified analyses further revealed that this association was particularly pronounced among fathers, consistent with previous studies (23–25). Although fathers are often less involved in routine childcare, they exert a disproportionate influence on family norms, dietary patterns, and activity-related decision-making. Consequently, international interventions have increasingly recognized fathers as a critical yet underutilized target group, with father-focused programs demonstrating promising effects on children’s lifestyle behaviors (23). Our findings suggested that negative paternal attitudes substantially undermine effective weight management practices, regardless of knowledge level. This phenomenon may reflect gendered social norms, whereby fathers’ beliefs are less often challenged by health education efforts traditionally focused on mothers. Consequently, father-inclusive and father-specific intervention strategies are not merely supplementary but essential for effective family-based obesity prevention. From a Family Systems Theory perspective, this pattern may indicate that fathers’ attitudes operate not only at the individual level but also within an interdependent family system, in which one parent’s beliefs and behaviors may shape shared routines, parenting practices, and the responses of other family members. The stronger association observed among fathers may also be interpreted through the lens of gendered socialization: culturally patterned expectations about paternal authority, food provision, physical activity, and caregiving roles may give fathers’ weight-related beliefs disproportionate influence over family-level decisions, even when mothers undertake more routine childcare. These theoretical perspectives extend the Social Cognitive Theory explanation by suggesting that paternal influence may occur through both behavioral modeling and the organization of family norms and roles.

The central role of attitude was confirmed through mediation analysis: the direct effect of parental weight management knowledge on practice was not significant, with attitude mediating 69.2% of the total effect. This result suggests that the mere acquisition of health knowledge by parents is insufficient to drive behavior change. Unless knowledge is internalized and transformed into positive, supportive attitudes, it is unlikely to translate into concrete actions that improve children’s weight-related outcomes. Empirical studies from diverse settings—including England, Europe, and East Asia—have similarly demonstrated that parental beliefs, perceived severity of obesity, and readiness to change exert stronger behavioral influence than factual knowledge alone (26–28). However, many prior studies relied on qualitative approaches or indirect inference. The distinctive contribution of our study lies in its quantitative estimation of mediation effects, offering concrete evidence that knowledge without attitudinal internalization is largely ineffective in driving behavioral change. Traditional perceptions that associate a larger body size with health, prosperity, or adequate caregiving remain prevalent, especially in multi-ethnic and economically transitioning regions. Consistent with a recent systematic review of Chinese adults’ perceptions of overweight and obesity (29), such culturally embedded beliefs may neutralize or even counteract the influence of biomedical knowledge. Our results thus reinforce the argument that effective interventions must explicitly address value systems, risk perceptions, and emotional responses, rather than relying solely on health education.

Additionally, this study observed that parents who were unaware of the weight management policy exhibited nearly twice the risk of poor practices compared with those who were aware, suggesting that policy promotion and dissemination are essential. Notably, stratified analyses revealed that the adverse effect of policy unawareness was particularly pronounced among urban parents and parents of overweight or obese children. These findings imply that among groups who ostensibly have greater access to information or a heightened need for intervention, failure of policy messaging to effectively reach them may pose an especially serious risk. Internationally, similar patterns have been observed in policy-driven obesity prevention initiatives (30, 31). Studies indicate that policy effectiveness depends not merely on dissemination but on parents’ perceived relevance and trust in the message source (31, 32). Our findings suggest that even in resource-rich urban settings, policy communication must be tailored and reinforced to translate into daily parenting decisions.

Moreover, rural residence emerged as an important risk factor for poor parental practices, supporting the notion that obesity burden is increasingly shifting toward rural areas amid rapid nutritional transition. Similar trends have been reported in rural communities in the United States and other middle-income countries, where increased availability of energy-dense foods coincides with limited health literacy and fewer supportive environments for physical activity (33). Furthermore, parents’ own uncontrolled eating behaviors were significantly associated with poor practices, underscoring the intergenerational transmission of dietary habits. This finding resonates strongly with Social Cognitive Theory, which emphasizes modeling and observational learning (34). Children tend to mirror parental eating patterns, suggesting that interventions targeting children alone are unlikely to succeed without parallel behavior change among parents.

Taken together, these findings provide a strong theoretical and empirical foundation for future intervention strategies. Given that attitude represents the central nexus between knowledge and practice, interventions should extend beyond conventional health education approaches. Importantly, our data on parents’ preferred sources of health information offer actionable insights. A majority of parents favored school-based lectures (76.7%), followed by educational materials from authoritative institutions (49.3%) and guidance from healthcare professionals (41.4%). This pattern suggests that a “school–family–medicine” collaborative model may be particularly effective in resource-limited settings.

Several limitations of this study should be acknowledged. First, the cross-sectional design precludes causal inference. Second, convenience sampling may have introduced selection bias and limits the representativeness of the sample. However, balance assessment indicated good comparability between the positive- and negative-practice groups, and additional analyses using PSOW weighting yielded results consistent with the primary findings, supporting the robustness of our conclusions. Second, the reliance on self-reported data introduces the possibility of social desirability bias, which may have influenced the KAP results. Parents might have overreported favorable knowledge, attitudes, or practices, potentially leading to inflated estimates (35). Third, despite identifying several significant correlates, this study did not account for all potential confounding variables. Factors such as the number of children in the household and total household size, which may influence parental capacity and resource allocation for weight management, were not included in the analysis. Future studies should use more representative sampling and, where multiple survey waves are available, longitudinal designs to clarify temporal relationships and assess the stability of the observed mediation pathways; incorporation of objective measures of child weight-related behaviors and outcomes would further strengthen the evidence.

## Conclusion

In conclusion, this study revealed that parental attitude functions as the critical bridge between knowledge and practice, particularly among fathers and overweight or obese children. Our results provided a robust basis for developing childhood obesity interventions in China, particularly in its resource-constrained central and western regions. These interventions can go beyond traditional knowledge dissemination and emphasize precision policy implementation and context-specific adaptation. Future public health efforts should prioritize strategies that actively engage and reshape parental attitudes, thereby translating national policy frameworks into sustained, family-level actions that protect and promote children’s health.

## Data Availability

The raw data supporting the conclusions of this article will be made available by the authors, without undue reservation.
