# Supplementary material for: Knowledge, attitudes, and practices of parents regarding childhood weight management and associated factors in Southwest China: a cross-sectional study
Source: Front Public Health. 2026 Sep 24;14:1907801. doi: 10.3389/fpubh.2026.1907801 (PMC13646047; doi:10.3389/fpubh.2026.1907801)
Supplement: Supplementary file 1 [file Supplementary_file_1.docx]

| **Table S1. Factors Associated with Poor Weight Management Practices Among Parents, After PSOW Weighting** | |
| --- | --- |
| **Variables** | **PSOW-Weighted**^a^ |
|  | OR(95% CI)P |
| **Uncontrolled eating** |  |
| No | ref |
| Yes | 1.77 (1.15–2.73)0.010 |
| **Awareness of China's 2024 weight management policy** |  |
| Yes | ref |
| No | 1.90 (1.22–2.96)0.005 |
| **Knowledge** |  |
| Adequate |  |
| Inadequate | 1.19 (0.73–1.94)0.477 |
| **Attitude** |  |
| Positive | ref |
| Negative | 5.78 (3.55–9.40)<0.001 |
| ^a^Adjusted for child characteristics (age, sex, current BMI status, chronic disease) and parent characteristics (age, sex, ethnicity, residence, education, occupation, family monthly income, chronic disease). | |

| **Table S2. Factors Associated with Poor Weight Management Practices among Parents Aged 18-35 Years** | | |
| --- | --- | --- |
| **Variables** | **Univariable** | **Multivariable** |
|  | cOR(95% CI)P | aOR(95% CI)P |
| **Child Characteristics** |  |  |
| **Age (years)** |  |  |
| 6-7 | ref |  |
| 8-9 | 1.07(0.49-2.56)0.864 |  |
| 10-12 | 1.16(0.52-2.77)0.729 |  |
| **Sex** |  |  |
| Male | ref |  |
| Female | 0.77(0.48-1.22)0.277 |  |
| **BMI status (parent-reported)** |  |  |
| Underweight | ref |  |
| Normal weight | 0.93(0.55-1.59)0.787 |  |
| Overweight or obese | 0.79(0.42-1.48)0.462 |  |
| **Chronic disease** |  |  |
| No | ref |  |
| Yes | 1.38(0.6-2.98)0.422 |  |
| **Parent Characteristics** |  |  |
| **Sex** |  |  |
| Male | ref |  |
| Female | 0.69(0.42-1.16)0.155 |  |
| **Ethnic**ity |  |  |
| Han | ref |  |
| Minority | 0.96(0.4-2.55)0.925 | 1.88(0.64-6.02)0.268 |
| Other | 0.54(0.19-1.59)0.241 | 0.87(0.26-3.07)0.82 |
| **Residence** |  | |
| Urban | ref |  |
| Rural | 1.47(0.9-2.45)0.128 | 2.31(1.26-4.37)0.008 |
| **Education** |  |  |
| Primary school and below | ref |  |
| Junior school | 1.22(0.47-3.57)0.697 |  |
| High school | 0.98(0.36-3.02)0.975 |  |
| College and above | 0.98(0.38-2.87)0.972 |  |
| **Occupation** |  |  |
| Enterprise worker | ref |  |
| Government employee | 0.49(0.23-1.04)0.066 |  |
| Self-employed | 0.72(0.37-1.4)0.326 |  |
| Unemployed | 0.2(0.03-0.77)0.04 |  |
| Farmer | 0.45(0.21-0.96)0.04 |  |
| Other | 0.88(0.34-2.21)0.789 |  |
| **Family income (CNY/month)** |  |  |
| ＜2,000 | ref | ref |
| 2,001~3,000 | 1.18(0.62-2.28)0.624 | 1.25(0.6-2.69)0.553 |
| 3,001~4,999 | 1.52(0.81-2.9)0.199 | 1.61(0.76-3.51)0.22 |
| ≥5,000 | 0.65(0.29-1.41)0.279 | 0.6(0.23-1.52)0.285 |
| **Chronic disease** |  |  |
| No | ref |  |
| Yes | 1.0(0.35-2.48)0.994 |  |
| **Cognitive restraint eating** |  |  |
| No | ref |  |
| Yes | 0.88(0.55-1.39)0.586 |  |
| **Emotional eating** |  |  |
| No | ref |  |
| Yes | 0.78(0.49-1.23)0.279 |  |
| **Uncontrolled eating** |  |  |
| No | ref | ref |
| Yes | 1.57(1-2.48)0.052 | 1.74(1.05-2.92)0.034 |
| **Awareness of China's 2024 weight management policy** |  |  |
| Yes | ref | ref |
| No | 2.14(1.35-3.44)0.001 | 2.0(1.18-3.42)0.011 |
| **Knowledge** |  |  |
| Adequate | ref |  |
| Inadequate | 1.98(1.25-3.15)0.003 |  |
| **Attitude** |  |  |
| Positive | ref | ref |
| Negative | 6.02(3.63-10.09)<0.001 | 7.49(4.28-13.43)<0.001 |

| **Table S3. Factors Associated with Poor Weight Management Practices among Parents Aged ≥35 Years** | | |
| --- | --- | --- |
| **Variables** | **Univariable** | **Multivariable** |
|  | cOR(95% CI)P | aOR(95% CI)P |
| **Child Characteristics** |  |  |
| **Age (years)** |  |  |
| 6-7 | ref |  |
| 8-9 | 0.7(0.32-1.56)0.377 |  |
| 10-12 | 0.54(0.21-1.33)0.184 |  |
| **Sex** |  |  |
| Male | ref | ref |
| Female | 1.37(0.7-2.7)0.357 | 1.95(0.93-4.25)0.082 |
| **BMI status (parent-reported)** |  |  |
| Underweight | ref |  |
| Normal weight | 0.58(0.23-1.54)0.26 |  |
| Overweight or obese | 1.46(0.59-3.8)0.42 |  |
| **Chronic disease** |  |  |
| No | ref |  |
| Yes | 1.34(0.4-3.92)0.605 |  |
| **Parent Characteristics** |  |  |
| **Sex** |  |  |
| Male | ref |  |
| Female | 0.82(0.38-1.87)0.621 |  |
| **Ethnic**ity |  |  |
| Han | ref |  |
| Minority | 1.16(0.53-2.67)0.717 |  |
| Other | 1.31(0.44-3.78)0.619 |  |
| **Residence** |  | |
| Urban | ref |  |
| Rural | 0.94(0.48-1.91)0.867 |  |
| **Education** |  |  |
| Primary school and below | ref |  |
| Junior school | 0.47(0.11-2.22)0.325 |  |
| High school | 0.35(0.08-1.62)0.164 |  |
| College and above | 0.42(0.1-1.84)0.229 |  |
| **Occupation** |  |  |
| Enterprise worker | ref |  |
| Government employee | 1.08(0.28-3.87)0.903 |  |
| Self-employed | 1.34(0.54-3.59)0.544 |  |
| Unemployed | 1.18(0.27-4.63)0.814 |  |
| Farmer | 1.18(0.27-4.63)0.814 |  |
| Other | 0.77(0.21-2.68)0.689 |  |
| **Family income (CNY/month)** |  |  |
| ＜2,000 | ref |  |
| 2,001~3,000 | 1(0.37-2.87)1 |  |
| 3,001~4,999 | 0.44(0.14-1.35)0.147 |  |
| ≥5,000 | 0.57(0.2-1.69)0.303 |  |
| **Chronic disease** |  |  |
| No | ref |  |
| Yes | 1.77(0.35-7.53)0.446 |  |
| **Cognitive restraint eating** |  |  |
| No | ref |  |
| Yes | 0.67(0.33-1.31)0.247 |  |
| **Emotional eating** |  |  |
| No | ref |  |
| Yes | 0.86(0.44-1.68)0.666 |  |
| **Uncontrolled eating** |  |  |
| No | ref | ref |
| Yes | 1.62(0.82-3.18)0.165 | 1.96(0.92-4.23)0.083 |
| **Awareness of China's 2024 weight management policy** |  |  |
| Yes | ref | ref |
| No | 2.37(1.21-4.71)0.012 | 2.09(1-4.39)0.05 |
| **Knowledge** |  |  |
| Adequate | ref |  |
| Inadequate | 2.09(1.05-4.14)0.034 |  |
| **Attitude** |  |  |
| Positive | ref | ref |
| Negative | 4.14(1.94-8.92)<0.001 | 4.3(1.91-9.91)<0.001 |

| **Table S4. Factors Associated with Poor Weight Management Practices among Fathers** | | |
| --- | --- | --- |
| **Variables** | **Univariable** | **Multivariable** |
|  | cOR(95% CI)P | aOR(95% CI)P |
| **Child Characteristics** |  |  |
| **Age (years)** |  |  |
| 6-7 | ref | ref |
| 8-9 | 0.68(0.25-1.94)0.455 | 0.13(0.02-0.62)0.014 |
| 10-12 | 0.69(0.23-2.08)0.5 | 0.15(0.03-0.71)0.019 |
| **Sex** |  |  |
| Male | ref |  |
| Female | 0.71(0.29-1.65)0.444 |  |
| **BMI status (parent-reported)** |  |  |
| Underweight | ref |  |
| Normal weight | 0.73(0.29-1.84)0.501 |  |
| Overweight or obese | 1.87(0.75-4.79)0.185 |  |
| **Chronic disease** |  |  |
| No | ref |  |
| Yes | 2.41(0.63-9.13)0.185 |  |
| **Parent Characteristics** |  |  |
| **Age (years)** |  |  |
| 18-34 | ref |  |
| ≥35 | 1.14(0.51-2.65)0.761 |  |
| **Ethnic**ity |  |  |
| Han | ref |  |
| Minority | 0.86(0.24-3.48)0.822 |  |
| Other | 0.44(0.09-2.19)0.303 |  |
| **Residence** |  | |
| Urban | ref |  |
| Rural | 0.89(0.42-1.93)0.755 |  |
| **Education** |  |  |
| Primary school and below | ref |  |
| Junior school | 0.78(0.2-3.15)0.723 |  |
| High school | 0.34(0.08-1.46)0.141 |  |
| College and above | 0.45(0.12-1.77)0.238 |  |
| **Occupation** |  |  |
| Enterprise worker | ref |  |
| Government employee | 0.31(0.09-1)0.056 |  |
| Self-employed | 0.47(0.17-1.27)0.135 |  |
| Unemployed | 6804687.93(0-NA)0.991 |  |
| Farmer | 0.45(0.12-1.63)0.237 |  |
| Other | 0.68(0.14-2.87)0.6 |  |
| **Family income (CNY/month)** |  |  |
| ＜2,000 | ref | ref |
| 2,001~3,000 | 3.17(1.01-10.92)0.055 | 9.71(2.27-49.53)0.003 |
| 3,001~4,999 | 1.65(0.54-5.42)0.39 | 3.7(0.89-17.59)0.082 |
| ≥5,000 | 0.79(0.25-2.67)0.696 | 1.31(0.29-6.24)0.729 |
| **Chronic disease** |  |  |
| No | ref |  |
| Yes | 2.79(0.45-53.56)0.351 |  |
| **Cognitive restraint eating** |  |  |
| No | ref |  |
| Yes | 1.24(0.6-2.58)0.566 |  |
| **Emotional eating** |  |  |
| No | ref |  |
| Yes | 0.87(0.42-1.85)0.719 |  |
| **Uncontrolled eating** |  |  |
| No | ref | ref |
| Yes | 4.12(1.93-9.23)<0.001 | 4.12(1.93-9.23)<0.001 |
| **Awareness of China's 2024 weight management policy** |  |  |
| Yes | ref |  |
| No | 2.76(1.3-6.12)0.01 |  |
| **Knowledge** |  |  |
| Adequate | ref | ref |
| Inadequate | 3.29(1.54-7.31)0.003 | 3.02(1.16-8.23)0.026 |
| **Attitude** |  |  |
| Positive | ref | ref |
| Negative | 5.11(2.33-11.53)<0.001 | 11.31(3.85-38.73)<0.001 |

| **Table S5. Factors Associated with Poor Weight Management Practices among Mothers** | | |
| --- | --- | --- |
| **Variables** | **Univariable** | **Multivariable** |
|  | cOR(95% CI)P | aOR(95% CI)P |
| **Child Characteristics** |  |  |
| **Age (years)** |  |  |
| 6-7 | ref |  |
| 8-9 | 0.92(0.49-1.81)0.804 |  |
| 10-12 | 0.93(0.48-1.85)0.828 |  |
| **Sex** |  |  |
| Male | ref |  |
| Female | 1.08(0.7-1.67)0.732 |  |
| **BMI status (parent-reported)** |  |  |
| Underweight | ref |  |
| Normal weight | 0.84(0.5-1.45)0.533 |  |
| Overweight or obese | 0.82(0.44-1.5)0.509 |  |
| **Chronic disease** |  |  |
| No | ref |  |
| Yes | 1.15(0.52-2.4)0.714 |  |
| **Parent Characteristics** |  |  |
| **Age (years)** |  |  |
| 18-34 | ref |  |
| ≥35 | 0.96(0.61-1.54)0.876 |  |
| **Ethnic**ity |  |  |
| Han | ref |  |
| Minority | 1.15(0.61-2.27)0.682 |  |
| Other | 0.87(0.39-1.94)0.73 |  |
| **Residence** |  | |
| Urban | ref | ref |
| Rural | 1.45(0.91-2.37)0.125 | 1.9(1.13-3.25)0.017 |
| **Education** |  |  |
| Primary school and below | ref |  |
| Junior school | 1.17(0.43-3.76)0.777 |  |
| High school | 1.09(0.38-3.59)0.883 |  |
| College and above | 1.08(0.4-3.45)0.881 |  |
| **Occupation** |  |  |
| Enterprise worker | ref |  |
| Government employee | 0.84(0.38-1.82)0.657 |  |
| Self-employed | 1.15(0.61-2.24)0.673 |  |
| Unemployed | 0.48(0.15-1.35)0.186 |  |
| Farmer | 0.69(0.32-1.5)0.352 |  |
| Other | 0.9(0.37-2.12)0.809 |  |
| **Family income (CNY/month)** |  |  |
| ＜2,000 | ref |  |
| 2,001~3,000 | 0.91(0.49-1.71)0.763 |  |
| 3,001~4,999 | 0.98(0.52-1.84)0.94 |  |
| ≥5,000 | 0.63(0.3-1.31)0.219 |  |
| **Chronic disease** |  |  |
| No | ref |  |
| Yes | 1.69(0.66-4.02)0.246 |  |
| **Cognitive restraint eating** |  |  |
| No | ref |  |
| Yes | 0.66(0.42-1.05)0.081 |  |
| **Emotional eating** |  |  |
| No | ref |  |
| Yes | 0.76(0.49-1.18)0.227 |  |
| **Uncontrolled eating** |  |  |
| No | ref |  |
| Yes | 1.13(0.72-1.75)0.598 |  |
| **Awareness of China's 2024 weight management policy** |  |  |
| Yes | ref | ref |
| No | 2.01(1.29-3.15)0.002 | 1.92(1.2-3.11)0.007 |
| **Knowledge** |  |  |
| Adequate | ref |  |
| Inadequate | 1.63(1.03-2.56)0.035 |  |
| **Attitude** |  |  |
| Positive | ref | ref |
| Negative | 5.35(3.25-8.87)<0.001 | 5.44(3.25-9.17)<0.001 |

| **Table S6. Factors Associated with Poor Weight Management Practices among Children Classified as Underweight by BMI** | | |
| --- | --- | --- |
| **Variables** | **Univariable** | **Multivariable** |
|  | cOR(95% CI)P | aOR(95% CI)P |
| **Child Characteristics** |  |  |
| **Age (years)** |  |  |
| 6-7 | ref |  |
| 8-9 | 0.66(0.21-2.32)0.487 |  |
| 10-12 | 1.1(0.34-3.93)0.876 |  |
| **Sex** |  |  |
| Male | ref |  |
| Female | 0.73(0.34-1.52)0.405 |  |
| **Chronic disease** |  |  |
| No | ref |  |
| Yes | 0.76(0.16-2.66)0.692 |  |
| **Parent Characteristics** |  |  |
| **Age (years)** |  |  |
| 18-34 | ref |  |
| ≥35 | 1.03(0.44-2.57)0.941 |  |
| **Sex** |  |  |
| Male | ref |  |
| Female | 0.9(0.41-2.06)0.801 |  |
| **Ethnic**ity |  |  |
| Han | ref |  |
| Minority | 0.93(0.24-4.53)0.917 |  |
| Other | 0.78(0.17-4.26)0.754 |  |
| **Residence** |  | |
| Urban | ref | ref |
| Rural | 1.52(0.72-3.36)0.283 | 5.04(1.69-17.11)0.006 |
| **Education** |  |  |
| Primary school and below | ref | ref |
| Junior school | 1.82(0.22-38.14)0.615 | 1.56(0.11-44.9)0.76 |
| High school | 0.48(0.05-10.85)0.562 | 0.2(0.01-6.81)0.311 |
| College and above | 1.14(0.13-24.01)0.916 | 1.73(0.09-60.55)0.728 |
| **Occupation** |  |  |
| Enterprise worker | ref | ref |
| Government employee | 0.24(0.03-1.16)0.101 | 0.08(0.01-0.59)0.019 |
| Self-employed | 1.02(0.36-3.05)0.978 | 2.24(0.57-9.57)0.259 |
| Unemployed | 0.71(0.03-6.81)0.786 | 0.85(0.03-11.12)0.905 |
| Farmer | 0.86(0.25-2.95)0.804 | 1.33(0.22-7.94)0.755 |
| Other | 0.86(0.18-3.65)0.837 | 2.84(0.48-16.91)0.242 |
| **Family income (CNY/month)** |  |  |
| ＜2,000 | ref |  |
| 2,001~3,000 | 1(0.37-2.82)0.994 |  |
| 3,001~4,999 | 0.72(0.25-2.07)0.534 |  |
| ≥5,000 | 0.7(0.2-2.33)0.566 |  |
| **Chronic disease** |  |  |
| No | ref |  |
| Yes | 1.53(0.22-30.37)0.71 |  |
| **Cognitive restraint eating** |  |  |
| No | ref |  |
| Yes | 0.63(0.28-1.36)0.251 |  |
| **Emotional eating** |  |  |
| No | ref | ref |
| Yes | 0.38(0.17-0.8)0.012 | 0.45(0.16-1.18)0.107 |
| **Uncontrolled eating** |  |  |
| No | ref |  |
| Yes | 0.93(0.44-1.94)0.851 |  |
| **Awareness of China's 2024 weight management policy** |  |  |
| Yes | ref |  |
| No | 1.43(0.68-3.07)0.35 |  |
| **Knowledge** |  |  |
| Adequate | ref | ref |
| Inadequate | 2.71(1.28-5.83)0.01 | 2.73(0.88-8.83)0.085 |
| **Attitude** |  |  |
| Positive | ref | ref |
| Negative | 5.5(2.45-12.68)<0.001 | 7.38(2.5-24.24)<0.001 |

| **Table S7. Factors Associated with Poor Weight Management Practices among Children Classified as Normal Weight by BMI** | | |
| --- | --- | --- |
| **Variables** | **Univariable** | **Multivariable** |
|  | cOR(95% CI)P | aOR(95% CI)P |
| **Child Characteristics** |  |  |
| **Age (years)** |  |  |
| 6-7 | ref |  |
| 8-9 | 2.98(0.96-13.12)0.092 |  |
| 10-12 | 2.44(0.78-10.79)0.168 |  |
| **Sex** |  |  |
| Male | ref |  |
| Female | 1.01(0.57-1.77)0.984 |  |
| **Chronic disease** |  |  |
| No | ref |  |
| Yes | 1.14(0.4-2.9)0.787 |  |
| **Parent Characteristics** |  |  |
| **Age (years)** |  |  |
| 18-34 | ref |  |
| ≥35 | 1.66(0.88-3.25)0.126 |  |
| **Sex** |  |  |
| Male | ref |  |
| Female | 1.04(0.53-2.15)0.909 |  |
| **Ethnic**ity |  |  |
| Han | ref |  |
| Minority | 1.12(0.51-2.67)0.783 |  |
| Other | 0.63(0.21-1.83)0.393 |  |
| **Residence** |  | |
| Urban | ref | ref |
| Rural | 1.85(0.99-3.61)0.063 | 2.58(1.29-5.45)0.01 |
| **Education** |  |  |
| Primary school and below | ref |  |
| Junior school | 0.75(0.25-2.58)0.628 |  |
| High school | 0.91(0.29-3.19)0.871 |  |
| College and above | 0.62(0.21-2.13)0.418 |  |
| **Occupation** |  |  |
| Enterprise worker | ref |  |
| Government employee | 0.51(0.17-1.4)0.2 |  |
| Self-employed | 0.81(0.36-1.84)0.603 |  |
| Unemployed | 0.6(0.15-2.01)0.426 |  |
| Farmer | 0.49(0.17-1.31)0.161 |  |
| Other | 1.14(0.41-3.07)0.802 |  |
| **Family income (CNY/month)** |  |  |
| ＜2,000 | ref |  |
| 2,001~3,000 | 0.99(0.46-2.16)0.972 |  |
| 3,001~4,999 | 0.82(0.37-1.84)0.628 |  |
| ≥5,000 | 0.46(0.18-1.14)0.099 |  |
| **Chronic disease** |  |  |
| No | ref | ref |
| Yes | 2.55(0.81-7.62)0.095 | 3.12(0.84-11.25)0.081 |
| **Cognitive restraint eating** |  |  |
| No | ref |  |
| Yes | 1.26(0.72-2.21)0.425 |  |
| **Emotional eating** |  |  |
| No | ref |  |
| Yes | 1.17(0.66-2.14)0.596 |  |
| **Uncontrolled eating** |  |  |
| No | ref | ref |
| Yes | 2.45(1.38-4.41)0.002 | 2.81(1.51-5.37)0.001 |
| **Awareness of China's 2024 weight management policy** |  |  |
| Yes | ref | ref |
| No | 2.54(1.43-4.62)0.002 | 1.98(1.05-3.8)0.036 |
| **Knowledge** |  |  |
| Adequate | ref |  |
| Inadequate | 1.44(0.81-2.55)0.215 |  |
| **Attitude** |  |  |
| Positive | ref | ref |
| Negative | 3.7(1.98-6.93)<0.001 | 3.74(1.89-7.49)<0.001 |

| **Table S8. Factors Associated with Poor Weight Management Practices among Children Classified as Overweight or Obese by BMI** | | |
| --- | --- | --- |
| **Variables** | **Univariable** | **Multivariable** |
|  | cOR(95% CI)P | aOR(95% CI)P |
| **Child Characteristics** |  |  |
| **Age (years)** |  |  |
| 6-7 | ref | ref |
| 8-9 | 0.52(0.23-1.17)0.111 | 0.39(0.13-1.11)0.083 |
| 10-12 | 0.42(0.15-1.1)0.082 | 0.26(0.06-0.95)0.05 |
| **Sex** |  |  |
| Male | ref | ref |
| Female | 1.06(0.53-2.12)0.867 | 2.12(0.8-5.88)0.137 |
| **Chronic disease** |  |  |
| No | ref | ref |
| Yes | 3.35(0.96-12.23)0.056 | 5.72(1.11-33.08)0.04 |
| **Parent Characteristics** |  |  |
| **Age (years)** |  |  |
| 18-34 | ref |  |
| ≥35 | 0.56(0.28-1.12)0.101 |  |
| **Sex** |  |  |
| Male | ref | ref |
| Female | 0.39(0.18-0.85)0.016 | 0.33(0.1-1.03)0.057 |
| **Ethnic**ity |  |  |
| Han | ref |  |
| Minority | 1.23(0.46-3.65)0.696 |  |
| Other | 0.86(0.24-3.12)0.817 |  |
| **Residence** |  | |
| Urban | ref |  |
| Rural | 0.64(0.31-1.34)0.234 |  |
| **Education** |  |  |
| Primary school and below | ref |  |
| Junior school | 0.72(0.18-3.22)0.651 |  |
| High school | 0.76(0.18-3.49)0.713 |  |
| College and above | 0.8(0.23-3.22)0.735 |  |
| **Occupation** |  |  |
| Enterprise worker | ref |  |
| Government employee | 0.98(0.34-2.86)0.973 |  |
| Self-employed | 0.89(0.33-2.48)0.818 |  |
| Unemployed | 0.19(0.01-1.24)0.139 |  |
| Farmer | 0.52(0.14-1.84)0.323 |  |
| Other | 0.19(0.01-1.24)0.139 |  |
| **Family income (CNY/month)** |  |  |
| ＜2,000 | ref | ref |
| 2,001~3,000 | 2.17(0.66-8.56)0.227 | 4.68(0.8-34.25)0.104 |
| 3,001~4,999 | 3.1(0.98-11.98)0.07 | 11.74(2.05-89.8)0.01 |
| ≥5,000 | 1.43(0.42-5.75)0.586 | 3.33(0.54-24.89)0.214 |
| **Chronic disease** |  |  |
| No | ref |  |
| Yes | 1.68(0.4-11.44)0.523 |  |
| **Cognitive restraint eating** |  |  |
| No | ref | ref |
| Yes | 0.5(0.22-1.05)0.077 | 0.37(0.11-1.07)0.078 |
| **Emotional eating** |  |  |
| No | ref |  |
| Yes | 0.99(0.5-1.98)0.979 |  |
| **Uncontrolled eating** |  |  |
| No | ref | ref |
| Yes | 1.41(0.69-2.85)0.345 | 2.28(0.82-6.54)0.115 |
| **Awareness of China's 2024 weight management policy** |  |  |
| Yes | ref | ref |
| No | 2.74(1.36-5.62)0.005 | 3.59(1.42-9.65)0.009 |
| **Knowledge** |  |  |
| Adequate | ref |  |
| Inadequate | 2.6(1.29-5.33)0.008 |  |
| **Attitude** |  |  |
| Positive | ref | ref |
| Negative | 9.71(4.3-23.14)<0.001 | 13.11(4.9-39.49)<0.001 |

| **Table S9. Factors Associated with Poor Weight Management Practices in Urban Parent** | | |
| --- | --- | --- |
| **Variables** | **Univariable** | **Multivariable** |
|  | cOR(95% CI)P | aOR(95% CI)P |
| **Child Characteristics** |  |  |
| **Age (years)** |  |  |
| 6-7 | ref | ref |
| 8-9 | 0.66(0.26-1.71)0.374 | 0.16(0.04-0.56)0.004 |
| 10-12 | 0.41(0.15-1.14)0.082 | 0.07(0.02-0.28)<0.001 |
| **Sex** |  |  |
| Male | ref |  |
| Female | 1.08(0.55-2.1)0.833 |  |
| **BMI status (parent-reported)** |  |  |
| Underweight | ref |  |
| Normal weight | 0.69(0.3-1.59)0.374 |  |
| Overweight or obese | 1.8(0.77-4.29)0.178 |  |
| **Chronic disease** |  |  |
| No | ref |  |
| Yes | 1.35(0.49-3.36)0.533 |  |
| **Parent Characteristics** |  |  |
| **Age (years)** |  |  |
| 18-34 | ref |  |
| ≥35 | 0.75(0.38-1.52)0.42 |  |
| **Sex** |  |  |
| Male | ref |  |
| Female | 0.75(0.38-1.52)0.42 |  |
| **Ethnic**ity |  |  |
| Han | ref |  |
| Minority | 4.31(1.19-27.73)0.055 |  |
| Other | 2.6(0.56-18.53)0.26 |  |
| **Education** |  |  |
| Primary school and below | ref |  |
| Junior school | 0.89(0.28-3.45)0.857 |  |
| High school | 1.08(0.29-4.55)0.91 |  |
| College and above | 0.4(0.09-1.88)0.231 |  |
| **Occupation** |  |  |
| Enterprise worker | ref |  |
| Government employee | 0.27(0.04-1.4)0.148 |  |
| Self-employed | 0.37(0.13-1.09)0.069 |  |
| Unemployed | 0.24(0.03-1.24)0.116 |  |
| Farmer | 0.26(0.09-0.75)0.012 |  |
| Other | 0.51(0.12-1.95)0.333 |  |
| **Family income (CNY/month)** |  |  |
| ＜2,000 | ref |  |
| 2,001~3,000 | 1.21(0.54-2.76)0.639 |  |
| 3,001~4,999 | 1.17(0.46-2.88)0.738 |  |
| ≥5,000 | 0.55(0.12-1.93)0.391 |  |
| **Chronic disease** |  |  |
| No | ref |  |
| Yes | 2.47(0.44-46.5)0.4 |  |
| **Cognitive restraint eating** |  |  |
| No | ref |  |
| Yes | 0.97(0.49-1.91)0.936 |  |
| **Emotional eating** |  |  |
| No | ref |  |
| Yes | 1.1(0.57-2.19)0.772 |  |
| **Uncontrolled eating** |  |  |
| No | ref | ref |
| Yes | 1.7(0.87-3.36)0.125 | 2(0.88-4.72)0.102 |
| **Awareness of China's 2024 weight management policy** |  |  |
| Yes | ref | ref |
| No | 3.89(1.82-9.12)0.001 | 3.58(1.4-10.07)0.01 |
| **Knowledge** |  |  |
| Adequate | ref |  |
| Inadequate | 5.11(2.43-11.6)<0.001 | 3.52(1.38-9.78)0.011 |
| **Attitude** |  |  |
| Positive | ref | ref |
| Negative | 7.18(3.48-15.23)<0.001 | 7.16(2.96-18.43)<0.001 |

| **Table S10. Factors Associated with Poor Weight Management Practices in Rural Parent** | | |
| --- | --- | --- |
| **Variables** | **Univariable** | **Multivariable** |
|  | cOR(95% CI)P | aOR(95% CI)P |
| **Child Characteristics** |  |  |
| **Age (years)** |  |  |
| 6-7 | ref |  |
| 8-9 | 0.96(0.49-1.94)0.909 |  |
| 10-12 | 1.16(0.59-2.35)0.679 |  |
| **Sex** |  |  |
| Male | ref |  |
| Female | 0.86(0.54-1.35)0.514 |  |
| **BMI status (parent-reported)** |  |  |
| Underweight | ref |  |
| Normal weight | 0.83(0.48-1.47)0.518 |  |
| Overweight or obese | 0.76(0.41-1.42)0.387 |  |
| **Chronic disease** |  |  |
| No | ref |  |
| Yes | 1.54(0.6-3.72)0.346 |  |
| **Parent Characteristics** |  |  |
| **Age (years)** |  |  |
| 18-34 | ref |  |
| ≥35 | 1.17(0.72-1.94)0.526 |  |
| **Sex** |  |  |
| Male | ref |  |
| Female | 0.86(0.51-1.46)0.557 |  |
| **Ethnic**ity |  |  |
| Han | ref |  |
| Minority | 0.73(0.38-1.44)0.345 |  |
| Other | 0.53(0.23-1.2)0.126 |  |
|  |  | |
|  |  |  |
|  |  |  |
| **Education** |  |  |
| Primary school and below | ref |  |
| Junior school | 1.04(0.36-3.29)0.942 |  |
| High school | 0.55(0.19-1.76)0.289 |  |
| College and above | 0.75(0.28-2.25)0.584 |  |
| **Occupation** |  |  |
| Enterprise worker | ref |  |
| Government employee | 0.78(0.38-1.6)0.489 |  |
| Self-employed | 1.2(0.64-2.29)0.572 |  |
| Unemployed | 0.55(0.15-1.68)0.323 |  |
| Farmer | 1.39(0.49-3.73)0.522 |  |
| Other | 0.9(0.36-2.14)0.809 |  |
| **Family income (CNY/month)** |  |  |
| ＜2,000 | ref |  |
| 2,001~3,000 | 0.98(0.46-2.13)0.958 |  |
| 3,001~4,999 | 0.89(0.43-1.9)0.751 |  |
| ≥5,000 | 0.55(0.25-1.22)0.135 |  |
| **Chronic disease** |  |  |
| No | ref |  |
| Yes | 1.81(0.69-4.52)0.207 |  |
| **Cognitive restraint eating** |  |  |
| No | ref | ref |
| Yes | 0.73(0.46-1.16)0.193 | 0.62(0.37-1.03)0.067 |
| **Emotional eating** |  |  |
| No | ref |  |
| Yes | 0.7(0.44-1.1)0.117 |  |
| **Uncontrolled eating** |  |  |
| No | ref | ref |
| Yes | 1.58(1-2.49)0.051 | 1.8(1.09-2.99)0.022 |
| **Awareness of China's 2024 weight management policy** |  |  |
| Yes | ref |  |
| No | 1.91(1.21-3.03)0.005 | 1.75(1.08-2.87)0.024 |
| **Knowledge** |  |  |
| Adequate | ref |  |
| Inadequate | 1.51(0.93-2.45)0.093 |  |
| **Attitude** |  |  |
| Positive | ref | ref |
| Negative | 4.83(2.87-8.2)<0.001 | 4.69(2.76-8.06)<0.001 |

| **Table S11. Knowledge dimension (n,%)** | | |
| --- | --- | --- |
| **Items** | **False** | **True** |
| K1: Obesity/Overweight is a disease | 194 (33.9) | 378 (66.1) |
| K2: The causes of obesity/overweight include genetic factors and environmental factors. | 232 (40.6) | 340 (59.4) |
| K3: Obesity/overweight is associated with an increased risk of various chronic diseases. | 187 (32.7) | 385 (67.3) |
| K4: Lifestyle interventions for obesity/overweight patients primarily include diet, exercise, and behavioral modifications. | 116 (20.3) | 456 (79.7) |
| K5: There is no need to supplement with micronutrients during medical nutritional weight loss. | 264 (46.2) | 308 (53.8) |
| K6: Dietary recommendations for weight management include strict control of oil intake, moderate restriction of refined grains and meats, and ensuring adequate consumption of vegetables, fruits, and dairy products. | 111 (19.4) | 461 (80.6) |
| K7: Weight management should take into account individual health conditions and preferences, and exercise choices should progress gradually. | 89 (15.6) | 483 (84.4) |
| K8: Behavioral aspects of weight management include daily recording of weight, diet, and exercise, as well as regular measurement of waist and hip circumference. | 136 (23.8) | 436 (76.2) |
| K9: Behavioral interventions for weight management do not include maintaining regular sleep patterns. | 176 (30.8) | 396 (69.2) |
| K10: Obesity/overweight can be cured by surgery alone. | 92 (16.1) | 480 (83.9) |
| K11: The clinical goal of weight management for overweight/obese patients does not include the prevention of obesity-related diseases. | 218 (38.1) | 354 (61.9) |

| **Table S12. Attitude dimension (n,%)** | | | | | |
| --- | --- | --- | --- | --- | --- |
| **Items** | **Strongly disagree** | **Disagree** | **Neutral** | **Agree** | **Strongly agree** |
| A1: Believe that maintaining a healthy weight is important for children's healthy growth. | 25 ( 4.4) | 10 ( 1.7) | 42 ( 7.3) | 152 (26.6) | 343 (60.0) |
| A2: Believe that actively learning knowledge related to weight management is important for maintaining children's health. | 14 ( 2.4) | 10 ( 1.7) | 77 (13.5) | 163 (28.5) | 308 (53.8) |
| A3: Believe that parents' behavioral habits are closely related to their children's weight status. | 5 ( 0.9) | 41 ( 7.2) | 69 (12.1) | 232 (40.6) | 225 (39.3) |
| A4: Believe that when a child is overweight/obese, active measures should be taken to help them adjust their weight. | 3 ( 0.5) | 4 ( 0.7) | 83 (14.5) | 174 (30.4) | 308 (53.8) |
| A5: Believe that abnormal weight in children at the current stage (such as underweight, overweight, or obesity) is just a normal part of growing up. | 86 (15.0) | 194 (33.9) | 127 (22.2) | 119 (20.8) | 46 ( 8.0) |
| A6: Believe that children can lose weight simply by eating less. | 105 (18.4) | 259 (45.3) | 110 (19.2) | 64 (11.2) | 34 ( 5.9) |
| A7: Believe that while taking weight-loss medication, there is no need to adjust diet, exercise, or behavioral habits. | 323 (56.5) | 138 (24.1) | 50 ( 8.7) | 32 ( 5.6) | 29 ( 5.1) |
| A8: Believe that they are confident in helping their children maintain a healthy weight. | 4 ( 0.7) | 58 (10.1) | 156 (27.3) | 234 (40.9) | 120 (21.0) |
| A9: Believe that implementing scientific weight management (such as adjusting diet and increasing physical activity) presents no difficulties in practical life. | 27 ( 4.7) | 186 (32.5) | 215 (37.6) | 107 (18.7) | 37 ( 6.5) |

| **Table S13. Practice dimension (n,%)** | | | | | |
| --- | --- | --- | --- | --- | --- |
| **Items** | **Never** | **Rarely** | **Sometimes** | **Often** | **Always** |
| P1: Regularly measures the child's weight (at least once a month). | 0 ( 0.0) | 26 ( 4.5) | 185 (32.3) | 198 (34.6) | 163 (28.5) |
| P2: Pays attention to balanced nutrition when preparing meals for the child (e.g., including vegetables, fruits, and high-quality protein). | 5 ( 0.9) | 9 ( 1.6) | 142 (24.8) | 186 (32.5) | 230 (40.2) |
| P3: Restricts the child's consumption of high-sugar foods and sugary drinks. | 27 ( 4.7) | 35 ( 6.1) | 114 (19.9) | 172 (30.1) | 224 (39.2) |
| P4: Restricts the child from eating high-fat foods. | 25 ( 4.4) | 38 ( 6.6) | 119 (20.8) | 190 (33.2) | 200 (35.0) |
| P5: Encourages the child to engage in regular physical exercise. | 2 ( 0.3) | 5 ( 0.9) | 126 (22.0) | 170 (29.7) | 269 (47.0) |
| P6: Accompanies the child in participating in physical exercise or outdoor activities. | 3 ( 0.5) | 13 ( 2.3) | 190 (33.2) | 164 (28.7) | 202 (35.3) |
| P7: Controls the amount of time the child spends on electronic devices each day (e.g., watching TV, playing on phones, or using computers). | 23 ( 4.0) | 11 ( 1.9) | 99 (17.3) | 157 (27.4) | 282 (49.3) |
| P8: Encourages the child to go to bed early every day. | 2 ( 0.3) | 6 ( 1.0) | 72 (12.6) | 184 (32.2) | 308 (53.8) |
| P9: Pays attention to information related to children's health management. | 4 ( 0.7) | 9 ( 1.6) | 194 (33.9) | 169 (29.5) | 196 (34.3) |
| P10: Uses food as a reward for the child. | 29 ( 5.1) | 111 (19.4) | 233 (40.7) | 117 (20.5) | 82 (14.3) |
| P11: Controls the child's behavior of eating before bedtime. | 11 ( 1.9) | 32 ( 5.6) | 158 (27.6) | 178 (31.1) | 193 (33.7) |

Fig S1. Preferred channels for receiving information on childhood weight management among parents
